# A Multi-Aperture Technique for Longitudinal Miniaturization of UWB 3 dB Dual-Layer SIW Coupler

**DOI:** 10.3390/s24113376

**Published:** 2024-05-24

**Authors:** Ahmad Bilal, Abdul Hadee, Yash H. Shah, Sohom Bhattacharjee, Choon Sik Cho

**Affiliations:** 1 Department of Smart Air Mobility, Korea Aerospace University, Goyang 10540, Gyeonggi-do, Republic of Korea; ahmadbilal@kau.kr (A.B.); hadee02@kau.kr (A.H.); yashshah@kau.kr (Y.H.S.); sohom24@kau.kr (S.B.); 2 Department of Electrical and Electronic Engineering, Korea Aerospace University, Goyang 10540, Gyeonggi-do, Republic of Korea

**Keywords:** directional coupler, 3 dB coupler, miniaturization, broad-wall coupling

## Abstract

Microwave couplers are used in large numbers in beamforming networks, and their miniaturization can lead to a significant size reduction in the overall phased array. While the miniaturization of 3 dB couplers in the transverse direction (width) has been given considerable attention in the literature, there is minimal to no information on reducing coupler length. This is because of the trade-off between aperture length, bandwidth and coupling strength. The Bethe–Hole theory requires adding multiple apertures in the longitudinal direction for wide bandwidth, thus increasing the device length. Another factor is the aperture size, which determines the coupling strength and puts additional strain on the compactness of a 3 dB coupler. Contrariwise, this paper proposes to merge two weak (and hence compact) coupling mechanisms to design a wideband 3 dB coupler. This is achieved by using a longitudinal rectangular slot and three cross-slots in the transverse direction. Because of weak coupling, the slot sizes are smaller than a conventional 3 dB coupler, hence yielding a device whose length is less than one guided wavelength ($\lambda_{g}$) without compromising the bandwidth. The presented coupler is 0.63 $\lambda_{g}$ in length, which is smaller than the state-of-the-art while maintaining a fractional bandwidth of 37% that is comparable to half-mode substrate integrated waveguide (HMSIW) couplers.

## 1. Introduction

A microwave coupler is a key component in wireless systems and is characterized by its bandwidth and size, among other parameters. In many emerging systems, such as multi-beam array antenna [1], Nolen/Butler/Blass matrix [2,3,4,5,6], frequency diverse array radar [7,8] or load modulated balanced amplifier [9], microwave couplers are required in large numbers, and their miniaturization can lead to a significant size reduction in the overall sensing systems, such as radars, LiDARs and other test equipment such as vector network analyzers and radio communication testers.

Depending on the stacking of the primary and coupled waveguides, there exist two fundamental topologies of waveguide couplers. The structure is said to be ‘broad-wall’ if the two waveguides are stacked on top of each other and a coupling aperture is etched in the common broad-wall [10]. On the other hand, the term ‘narrow-wall’ is used when the design is realized on a single layer where two waveguides are coupled through an aperture in the side wall [11]. Out of these two, broad-wall coupling is a more compact approach, which yields a device that is almost half the width of its narrow-wall counterpart and is incorporated in this work.

Progress in substrate integrated waveguide (SIW) technology has paved the way towards further width reduction with the advent of half-mode SIW (HMSIW), which has been immensely utilized with different variations [12,13,14,15,16,17,18]. By bisecting the conventional SIW through the fictitious magnetic wall, Bing Liu et al. [12] have shown that coupler width can be reduced to half. A compact Butler matrix has recently been designed by Lei-Lei Qiu et al. [13] where they have used rectangular slot aperture on dual-layer HMSIW. In order to reduce coupler length, one method has recently been published by Qiang Sun et al. [1], where they have proposed to use an interdigital slot that is a manifestation of metamaterials. Their technique is applicable to narrow-wall couplers and yields a fractional bandwidth (FBW) of 13.3%. Another technique has been proposed by Shui Liu et al. [14], where they have used the wide-bandwidth property of HMSIW and reduced the number of Bethe–Hole apertures on a dual-layer HMSIW. Their design uses five Bethe–Hole apertures (1.39 $\lambda_{g}$) to achieve 34.5% FBW. Further variants of HMSIW incorporate the spoof surface plasmon polariton (SSPP) structure in the aperture to harness additional benefits. For instance, Hong-wei Deng et al. [15] have developed a balanced high common-mode suppression HMSIW coupler using SSPP. The slow wave property of SSPP is utilized by Ze-Ming Wu et al. [16] to reduce the coupler size and yield 18.6% FBW. A new slow-wave structure has been proposed by Yunfan Peng et al. [17] that reduces the phase velocity in HMSIW by 66.7%. They have designed a coupler based on this structure that yields 28.32% FBW with a length of just 0.517 $\lambda_{g}$. In addition to these methods, other techniques have also been developed, such as connecting the magnetic walls with metal strips [18], using air-filled vias to manipulate the effective permittivity [19] and using optimization algorithms to design the coupling structure [20]. Among these methods, only slow wave-based approaches look promising in reducing the length of a coupler.

This paper explicitly targets the longitudinal miniaturization, which has not been given due attention mainly because coupler length is directly proportional to its coupling strength and FBW. Whether it be a Bethe–Hole coupler or a branch-line hybrid [21], the typical method to increase FBW is to introduce multiple apertures/sections along the device length. Contrary to this traditional approach, there exists another less explored method that increases the coupling coefficient by placing the apertures in the transverse direction [22,23]. This method is utilized by Ali A et al. [22] by etching two parallel slot apertures to design a 6 dB coupler with 16% FBW. Shui Liu et al. [23] have considered this method in more detail and have used two rows of Bethe–Hole apertures to yield 3 dB coupling with 35.3% FBW and an aperture length of 1.3 $\lambda_{g}$. These results look promising, but one expects a smaller length since the voltage coupling coefficient is doubled because of the two aperture rows and not because of the aperture length. Both aperture rows are identical in these cases and have an equal contribution towards the coupled signal.

To reduce the coupler length without compromising the FBW and coupling strength, we propose a novel multi-aperture technique that is based on merging weak coupling coefficients of two non-identical compact aperture rows. The fundamental idea is to efficiently utilize the broad-wall area [24,25]. This method uses multiple weak but wideband coupling apertures whose coupling levels are carefully tuned to yield a 3 dB coupler with reduced length. We first design a traditional rectangular slot aperture coupler, but instead of achieving all coupling from a single aperture, we reduce its length and compromise on coupling level. Then, to compensate, three cross-slot apertures are designed whose coupling is even weaker. When both of these structures are placed on a common broad-wall, the field components superimpose, resulting in stronger coupling. In this paper, this principle is extensively analyzed through super-position of H-fields and coupling levels, and a stepwise design method is given with S-parameter plots at each stage. Furthermore, experimental validation is provided by designing a dual-layer SIW coupler, and the measured results are presented and compared with the state-of-the-art. It is shown that, because of the smaller contributions from individual apertures, the resulting device is smaller in size (0.63 $\lambda_{g}$) while benefiting from the wider FBW (37%) of the chosen aperture shapes and still yielding strong 3 dB coupling.

The rest of this article is organized as follows. Section 2 presents the theoretical background that leads to the proposed method. Section 3 shows the methodology adopted to design the coupling cavity. A GCPW-to-SIW transition is designed to feed the dual-layer SIW coupling cavity. The final device is manufactured, and the measurement results are shown in Section 4. Section 5 concludes this paper.

## 2. Theoretical Formulation

**Figure 1:**
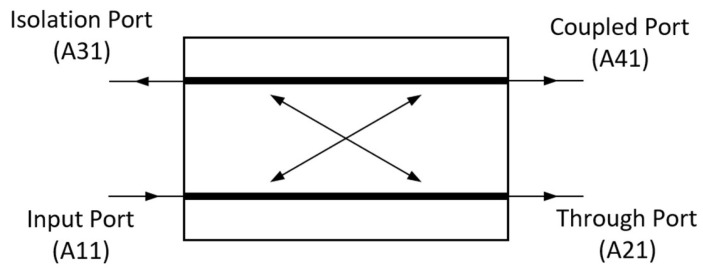
Schematic diagram of a coupler. The stated port names and numbers are followed throughout this article.

Figure 1 shows the schematic of a coupler with the port names and numbers that are followed in this article.

**Figure 2:**
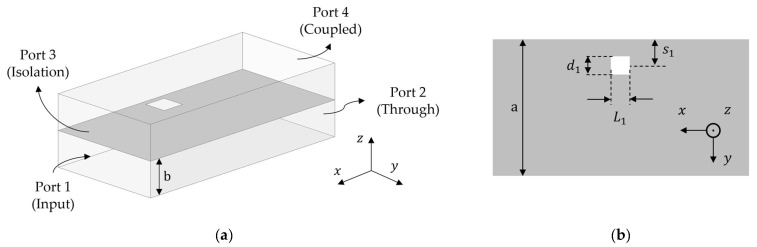
Geometry and coordinate system of the coupler. (**a**) Vertically stacked waveguides and (**b**) broad-wall with a slotted aperture.

We implement this by using two vertically stacked waveguides that have an aperture in the common broad-wall. The geometry along with the coordinate system and the broad-wall are shown in Figure 2.

### 2.1. Computation of Signal in Coupled Waveguide

The primary (bottom) and coupled (top) waveguides, which are separated by the broad-wall, are shown in Figure 2a. The two waveguides are identical with thickness b. The top view of the broad-wall is shown in Figure 2b. The shape, size and position of this aperture determine the bandwidth, coupling level and size of the coupler. Any arbitrarily shaped aperture can be chosen as long as its electric and magnetic polarizabilities are known. We use this geometry to calculate the signal in the coupled waveguide. The following steps outline this.

1. Excite the primary waveguide.

The primary waveguide is excited with TE_10_ mode at the input port. For the coordinate system shown in Figure 2, there exist $H_{x}$, $H_{y}$ and $E_{z}$ field components that are given by the following equations:

(1) $$H_{x}=j\frac{\pi }{ak_{o}\eta_{o}}\mathrm{Cos}\left(\frac{\pi y}{a}\right)e^{j\beta x}$$

(2) $$H_{y}=-\frac{\beta }{k_{o}\eta_{o}}\mathrm{Sin}\left(\frac{\pi y}{a}\right)e^{j\beta x}$$

(3) $$E_{z}=\mathrm{Sin}\left(\frac{\pi y}{a}\right)e^{j\beta x}$$

where $\beta =\sqrt{{k_{o}}^{2}-{k_{c}}^{2}}$ and $k_{o}=2\pi /\lambda$ is the free space wavenumber. The wavenumber at cut-off frequency is given by $k_{c}=\pi /a$, and $\eta_{o}$ is the intrinsic impedance of free space. The E-field component is normal to the aperture, while the H-field components are tangential and are travelling in the −x direction.

2. Calculate the equivalent polarization currents at the aperture.

The field components excite equivalent electric and magnetic polarization currents at the aperture location, which are directly proportional to the corresponding field components and can be computed by the equivalence theorem [26]. The electric polarization current $P_{e}$ is given by

(4) $$P_{e}=\hat{z}\epsilon_{o}\alpha_{e}\mathrm{Sin}\left(\frac{\pi s_{1}}{a}\right)$$

while the x and y components of the magnetic polarization currents are given by

(5) $$P_{m_{x}}=-{\hat{x}\;\alpha }_{m}\frac{j\pi }{ak_{o}\eta_{o}}\mathrm{Cos}\left(\frac{\pi s_{1}}{a}\right)$$

(6) $$P_{m_{y}}={\hat{y}\alpha }_{m}\frac{\beta }{k_{o}\eta_{o}}\mathrm{Sin}\left(\frac{\pi s_{1}}{a}\right)$$

where $\left(\hat{x},\hat{y},\hat{z}\right)$ are the unit vectors for the coordinate system shown in Figure 2 and $\alpha_{e}$ and $\alpha_{m}$ are the electric and magnetic polarizabilities, respectively. For the aperture shown in Figure 2b, $\alpha_{e}=\alpha_{m}=\pi L_{1}{d_{1}}^{2}/16$.

3. Calculate the electric and magnetic current sources.

The aperture can be replaced by its equivalent polarization currents, and by using Maxwell’s equations, these polarization currents can be related to electric and magnetic current sources, which then radiate in the coupled waveguide to give the coupled and isolated port signals. The electric current source is given by $J=j\omega P_{e}$, and using Equation (4),

(7) $$J=\hat{z}\;j\omega \epsilon_{o}\alpha_{e}\mathrm{Sin}\left(\frac{\pi s_{1}}{a}\right)$$

while the magnetic current source is given by $M=j\omega \mu_{o}P_{m}$. Using Equations (5) and (6),

(8) $$M_{x}=-\hat{x}\;j\omega \mu_{o}{\;\alpha }_{m}\frac{j\pi }{ak_{o}\eta_{o}}\mathrm{Cos}\left(\frac{\pi s_{1}}{a}\right)$$

(9) My=y^ jωμo αmβkoηoSin⁡πs1a

4. Calculate the forward $\left(S_{41}\right)$ and backward $\left(S_{31}\right)$ travelling signals.

#### 2.1.1. Expression for $S_{41}$

Since both waveguides are identical, only TE_10_ mode exists in the coupled waveguide, which can be excited by the current sources $J,\;M_{x}$ and $M_{y}$. This is a simple step because these are point sources and their contributions can be calculated by carrying out the following integrals [27]:

(10) $$S_{41_{J}}=-\frac{k_{o}\eta_{o}}{ab\beta }\int {E_{z}}^{-}∙J\;dv$$

(11) $$S_{41_{M_{i}}}=\frac{k_{o}\eta_{o}}{ab\beta }\int {H_{i}}^{-}∙M_{i}\;dv$$

where ${E_{z}}^{-}$ is the E-field component of TE_10_ mode that is travelling in the +x direction in the coupled waveguide and ${H_{i}}^{-}$ is the $i^{th}$ component of the corresponding H-field. The integral is carried out for the entire volume of the coupled waveguide, which reduces to a single point in this case. Using Equation (7) in (10) and (8), (9) in (11), we compute the individual contributions of the three field components in the coupled waveguide.


(12) $$S_{41_{J}}=\frac{{-j\omega \epsilon_{o}\alpha_{e}k}_{o}\eta_{o}}{ab\beta }{\mathrm{Sin}}^{2}\left(\frac{\pi s_{1}}{a}\right)$$

(13) $$S_{41_{M_{x}}}=\frac{j\omega \mu_{o}\alpha_{m}}{ab\beta k_{o}\eta_{o}}{\left(k_{c}\right)}^{2}{\mathrm{Cos}}^{2}\left(\frac{\pi s_{1}}{a}\right)$$

(14) $$S_{41_{M_{y}}}=\frac{j\omega \mu_{o}\alpha_{m}}{ab}\left(\frac{\beta }{k_{o}\eta_{o}}\right){\mathrm{Sin}}^{2}\left(\frac{\pi s_{1}}{a}\right)$$

It can be observed from these equations that the electric component $S_{41_{J}}$ is 180° out of phase from the magnetic components $S_{41_{M_{x}}}$ and $S_{41_{M_{y}}}$, which are in phase with respect to each other. Hence, they tend to cancel each other’s effect. We analyze the contribution of each component by plotting Equations (12)–(14) for the simulation parameters listed in Table 1.

**Figure 3:**
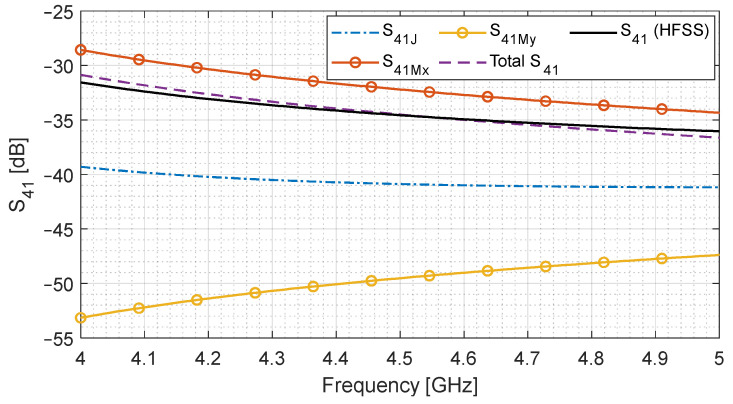
Contribution of electric and magnetic current sources towards the total coupled port signal ($S_{41}$). Total $S_{41}$ is compared with a full-wave simulation.

Figure 3 shows the magnitude of the coupled port signal as a function of frequency. It can be observed that the magnetic component $S_{41_{M_{x}}}$ has a much higher contribution as compared to $S_{41_{M_{y}}}$. Although $S_{41_{J}}$ is relatively high, it cancels the effect of $S_{41_{M_{x}}}$ since it is 180° out of phase. This is shown by the total $S_{41}$, which is lower than $S_{41_{M_{x}}}$ by a constant amount (since $S_{41_{J}}$ is approximately constant). Finally, the said geometry is simulated using HFSS to validate the analytical solution. The results are quite close, but the difference can be explained by the fact that the analytical solution models the aperture with a point source rather than a distribution of sources. However, this result is valid for initial design and analysis purposes.

#### 2.1.2. Expression for $S_{31}$

In Equations (10) and (11), by substituting the backward (+x) travelling components ${E_{z}}^{-}$ and ${H_{i}}^{-}$ with the forward (−x) travelling components ${E_{z}}^{+}$ and ${H_{i}}^{+}$, respectively, the signal at the isolated port ($S_{31}$) can be computed. The contributing components at the isolated port are given by the following equations:

(15) $$S_{31_{J}}=\frac{{-j\omega \epsilon_{o}\alpha_{e}k}_{o}\eta_{o}}{ab\beta }{\mathrm{Sin}}^{2}\left(\frac{\pi s_{1}}{a}\right)$$

(16) $$S_{31_{M_{x}}}=\frac{j\omega \mu_{o}\alpha_{m}}{ab\beta k_{o}\eta_{o}}{\left(k_{c}\right)}^{2}{\mathrm{Cos}}^{2}\left(\frac{\pi s_{1}}{a}\right)$$

(17) $$S_{31_{M_{y}}}=\frac{-j\omega \mu_{o}\alpha_{m}}{ab}\left(\frac{\beta }{k_{o}\eta_{o}}\right){\mathrm{Sin}}^{2}\left(\frac{\pi s_{1}}{a}\right)$$

**Figure 4:**
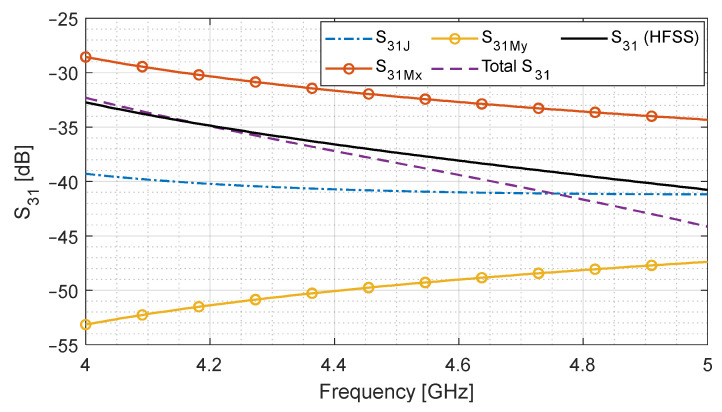
Contribution of electric and magnetic current sources towards the total isolated port signal ($S_{31}$). Total $S_{31}$ is compared with a full-wave simulation.

Since the forward and backward travelling $H_{y}$ components have opposite signs, $S_{31_{M_{y}}}$ goes out of phase as compared to $S_{41_{M_{y}}}$ in Equation (14). Now, it is in phase with $S_{31_{J}}$, hence contributing towards the cancellation of signals at the isolated port. We analyze the isolated port signals in Figure 4 by using the same geometry.

Figure 4 shows that the magnitude of all current sources is identical to Figure 3. However, the total $S_{31}$ is lower than $S_{41}$ because the contribution of magnetic current component $S_{31_{M_{y}}}$ is 180° out of phase with $S_{31_{M_{x}}}$ and, hence, cancels its effect. This is why the isolation signal is lower than the coupled port signal.

### 2.2. Effect of Geometric Parameters

In order to increase coupling, the straightforward method is to increase the aperture size, since we want more energy to transfer from the primary waveguide to the coupled waveguide. This is discussed in this subsection.

#### 2.2.1. Effect of Aperture Length and Width

**Figure 5:**
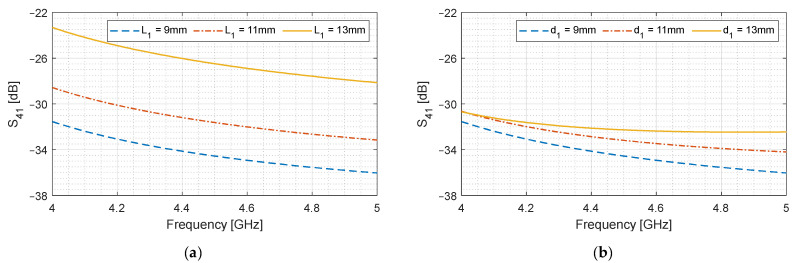
Effect of slot length and width on $S_{41}$. (**a**) Length $L_{1}$; (**b**) Width $d_{1}$.

We analyze the effect of increasing aperture length $L_{1}$ and width $d_{1}$ on $S_{41}$. We have observed that $H_{x}$ has the highest contribution, and from $J=\hat{n}\times H$, we know that the dimension orthogonal to $J_{y}$ should give the maximum increase in $S_{41}$. From the geometry of Figure 2b, $L_{1}$ is that dimension. The second highest contribution is from $E_{z}$ as shown in Figure 3, and increasing $d_{1}$ should also give some increase in $S_{41}$ since $E_{z}$ is maximum at the center of the waveguide. These effects are shown in Figure 5.

It can be observed in Figure 5a that increasing $L_{1}$ significantly increases $S_{41}$, while $d_{1}$ also has a slight impact. However, increasing $L_{1}$ implies that the coupler length must be increased in order to yield −3 dB coupling.

#### 2.2.2. Effect of Aperture Rotation

Since $J_{y}$ is orthogonal to the aperture length, it has the maximum contribution towards the coupled port signal. We can change the angle between $J_{y}$ and $L_{1}$ by rotating the aperture about its center. This rotation brings the effect of $J_{x}$ into play since it is no longer parallel to $L_{1}$. From Figure 3, we know that $J_{y}$ ($S_{41_{M_{x}}}$) causes $S_{41}$ to decrease with frequency, while $J_{x}$ ($S_{41_{M_{y}}}$) increases $S_{41}$ with frequency. Hence, by rotating the aperture, we can balance the effect of $J_{x}$ and $J_{y}$ and use it to stabilize the coupling level to a relatively constant value. This is critical because we have observed that there is a drastic change in coupling level with frequency in Figure 3 and Figure 5a. In practice, $S_{41}$ should be at −3 ± 0.5 dB across the entire bandwidth, and aperture rotation can help approach and stabilize at this level.

**Figure 6:**
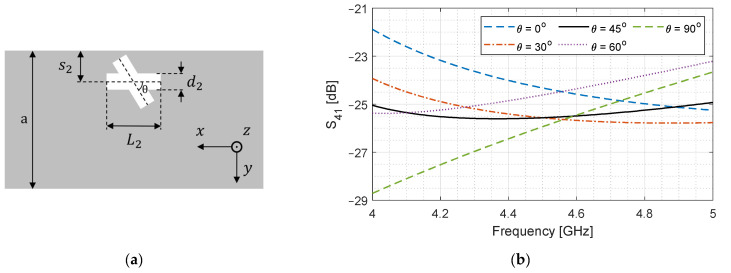
Effect of slot rotation angle θ on $S_{41}$. (**a**) Top view of broad-wall; (**b**) $S_{41}$ as a function of frequency.

Table 2 lists the simulation parameters to analyze the effect of aperture rotation. Figure 6a shows the broad-wall, while Figure 6b shows $S_{41}$ versus frequency when the aperture is rotated.

At θ=0°, it is just a longitudinal slot, and the coupling is decreasing with frequency, which is in line with Figure 3 and Figure 5a. As the slot is rotated to 30°, the change in $S_{41}$ reduces, and it becomes relatively stable at 45°. At θ=60°, $J_{x}$ becomes the dominant contributor, $S_{41}$ starts to increase with frequency, and the slope becomes maximum at 90°. We will use this effect in the subsequent sections to design the cross-slot aperture, which will enhance and stabilize $S_{41}$ to −3 ± 0.5 dB level.

### 2.3. Multi-Aperture Topologies

Another method to increase $S_{41}$ is by using multiple apertures, which can be etched along the longitudinal [14] or transverse directions [22,23] on the broad-wall.

#### 2.3.1. Apertures in the Longitudinal Direction

**Figure 7:**
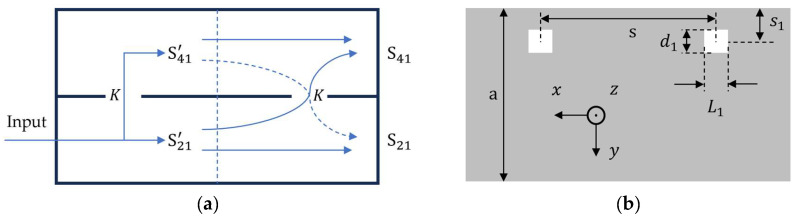
Coupled waveguides with two apertures. (**a**) Side view and (**b**) top view of broad-wall.

Figure 7 shows the waveguide coupler, where two identical apertures are etched along the longitudinal direction.

If we assume ideal operation, there is no signal travelling backward, and the forward travelling components are in phase from both apertures since they travel the same distance. In this case, ${\left|S_{21}^{\prime }\right|}^{2}+{\left|S_{41}^{\prime }\right|}^{2}=1$, and a coupling coefficient K can be defined as follows:

(18) $$K=\frac{\left|S_{41}^{\prime }\right|}{\left|S_{21}^{\prime }\right|}$$

where $S_{41}^{\prime }$ and $S_{21}^{\prime }$ are the coupled and through port signals from the first aperture as shown in Figure 7a. We can view this device as a series of two identical devices and write the $S_{41}$ of the whole device in terms of the S-parameters of only the first aperture. This can be performed by realizing that the total $S_{41}$ is the sum of the coupled component of $S_{21}^{\prime }$ through the second aperture $S_{21}^{\prime }K$ and the difference of $S_{41}^{\prime }$ and its coupled component $S_{41}^{\prime }K$. Mathematically, it can be written as $S_{41}=S_{21}^{\prime }K+(S_{41}^{\prime }-S_{41}^{\prime }K)$. Using Equation (18), this can be generalized for n apertures as follows:

(19) $$S_{41}=S_{41}^{\prime }{\left(2-K\right)}^{n-1}$$

For validation, we use HFSS to simulate $S_{41}^{\prime }$, use Equation (19) to compute $S_{41}$ and compare the results for a two-aperture coupler for the same geometric parameters as listed in Table 1 with the distance between the apertures s=5 mm.

**Figure 8:**
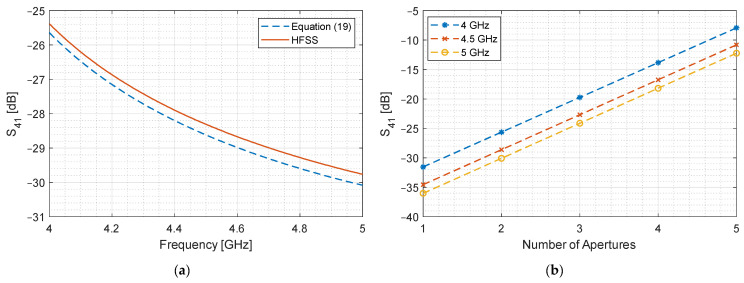
Evaluation of multi-aperture coupling. (**a**) $S_{41}$ as a function of frequency for n=2; (**b**) $S_{41}$ as a function of the number of apertures at 4, 4.5 and 5 GHz.

Figure 8a shows the coupled port signal as a function of frequency for a device with two identical apertures. A single-aperture device is simulated, and Equation (19) is used to evaluate the total coupling as if it were a two-aperture coupler. The result is compared with an actual two-aperture coupler, and a satisfactory match is found where the difference is below 0.4 dB. This validates Equation (19), which is then used to quantify the increment in coupling if we increase the number of apertures. An exponential rise is expected, which becomes linear on a logarithmic scale and is shown in Figure 8b. It can be observed that, in order to yield a −3 dB coupler, we need to increase the number of apertures, which increases its length. Secondly, there is a 4.5 dB difference in coupling between 4 GHz and 5 GHz. We need to reduce this difference to an acceptable level.

**Figure 9:**
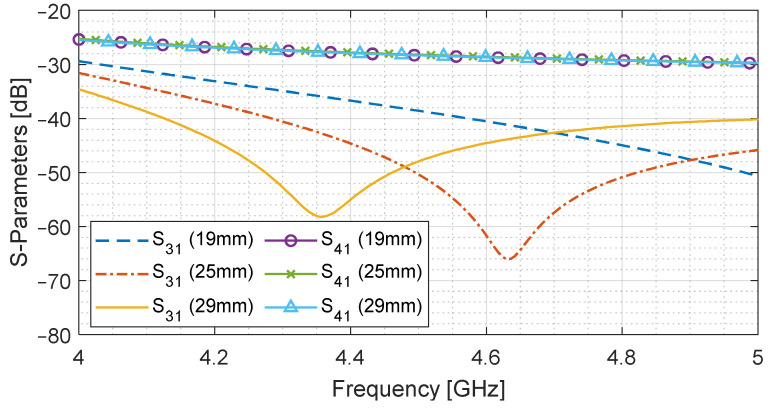
$S_{31}$ and $S_{41}$ as a function of frequency when the distance between the aperture is varied.

Since the forward-travelling signals from both apertures travel the same distance in order to reach the coupled port, the distance s between the apertures has no effect on $S_{41}$. However, the backward-travelling signal from the second aperture travels 2s more distance to reach the isolation port as compared to the signal coupled by the first aperture. Hence, $S_{31}$ changes with s. This effect is shown in Figure 9, where it can be observed that $S_{41}$ is unchanged when s is varied but $S_{31}$ dips down at certain frequencies for different values of s.

For instance, $S_{31}$ has a minimum value of −66 dB at 4.63 GHz when s=25 mm. This is because the backward signals from both apertures are out of phase at this frequency and cancel each other. This can be verified by computing the guided wavelength, which is given by

(20) $$\lambda_{g}=\frac{\lambda }{\sqrt{1-{\left(c/2af\right)}^{2}}}$$

for a waveguide operating in TE_10_ mode with $\epsilon_{r}=1.$ For the given waveguide parameters and f=4.63 GHz, the distance s between the apertures comes out to be approximately equal to $\lambda_{g}/4$. Hence, the backward signal, after travelling a distance of 2 s, becomes 180° out of phase and cancels the contribution by the first aperture at the isolation port, yielding a very small $S_{31}$. Although it improves the isolation, it should be noted that this phenomenon is frequency dependent, and more apertures are required to enhance the isolation bandwidth while increasing the coupler length [14].

#### 2.3.2. Apertures in the Transverse Direction

**Figure 10:**
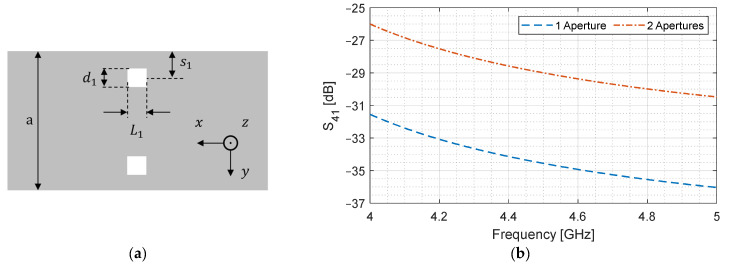
Effect of adding an aperture in the transverse direction. (**a**) Top view of broad-wall; (**b**) $S_{41}$ versus frequency.

It is intuitive to expect an increase in coupling if an aperture is added in the transverse direction. This is indeed the case since the current components across the magnetic wall go unhindered and can be used to enhance coupling. If we add an identical aperture across the y=0 plane, as shown in Figure 10a, it doubles the impedance that is posed by the aperture. This results in a doubling of the total voltage drop across the apertures, hence doubling the voltage coupling coefficient, which results in a 6 dB improvement in $S_{41}$ [23].

In Figure 10b, the $S_{41}$ of two apertures in the transverse direction is shown and is compared to the single-aperture case. There is an increase of 5.6 dB, which is very close to the true value. It should be noted that this increment can be changed if two non-identical apertures are used, since they would have different impedances. This result is important because adding an aperture in the transverse direction increases coupling without increasing the coupler length. This is unlike increasing the aperture length or increasing the number of apertures in the longitudinal direction. Hence, it can be used to engineer a device that is smaller in length while having −3 dB coupling.

We summarize the insights developed so far.

1. In broad-wall aperture coupling using TE_10_ mode, $H_{x}$ has the highest contribution towards $S_{41}$. The second highest contribution comes from $E_{z}$, while $H_{y}$ has very little offering.
2. Aperture length drastically increases $S_{41}$, while aperture width has a relatively low effect.
3. Rotating the aperture signifies the effect of $H_{y}$ while diminishing the effect of $H_{x}$. This can be used to reduce the fluctuation in $S_{41}$.
4. Multiple apertures along the broad-wall length exponentially increase coupling. The distance between apertures does not affect $S_{41}$, but it should be $\lambda_{g}/4$ for enhanced isolation.
5. Apertures in the transverse direction increase coupling by 6 dB.

## 3. Design Methodology

We use the methods discussed in the previous section to design a longitudinally compact UWB −3 dB coupler.

### 3.1. Longitudinal Slot Aperture

**Figure 11:**
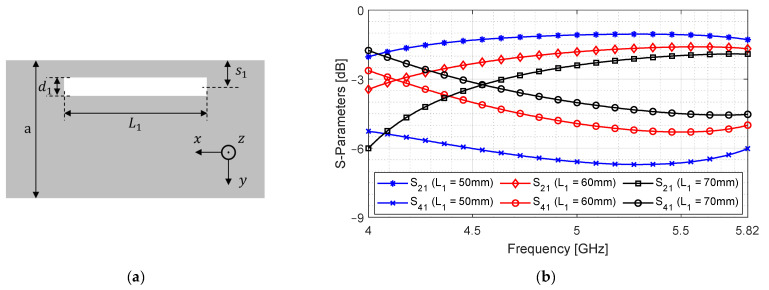
Design of longitudinal slot aperture. (**a**) Top view of broad-wall; (**b**) $S_{21}$ and $S_{41}$ versus frequency.

As discussed in the previous section, increasing aperture length maximizes the contribution of $J_{y}$. We first design a slot aperture and increase its length to a maximum such that $S_{41}$ could approach −3 dB. The broad-wall and the through and coupled port signals are shown in Figure 11 for various lengths $L_{1}$.

It can be observed in Figure 11b that, as the length increases, more signal reaches the coupled port, which in turn reduces $S_{21}$. We can continue increasing $L_{1}$, but there are two problems with this approach. Firstly, it increases the device length, and secondly, it limits the bandwidth for which $S_{21}$ and $S_{41}$ remain within ±0.5 dB of each other. This makes it difficult to design a −3 dB coupler that has a wide bandwidth. We can counter both issues by settling for a smaller length with reduced coupling and obtain additional coupling from the transverse direction. In this case, we limit $L_{1}$ to 53 mm, which gives a suitable compromise between the average magnitude and fluctuation of $S_{41}$.

**Figure 12:**
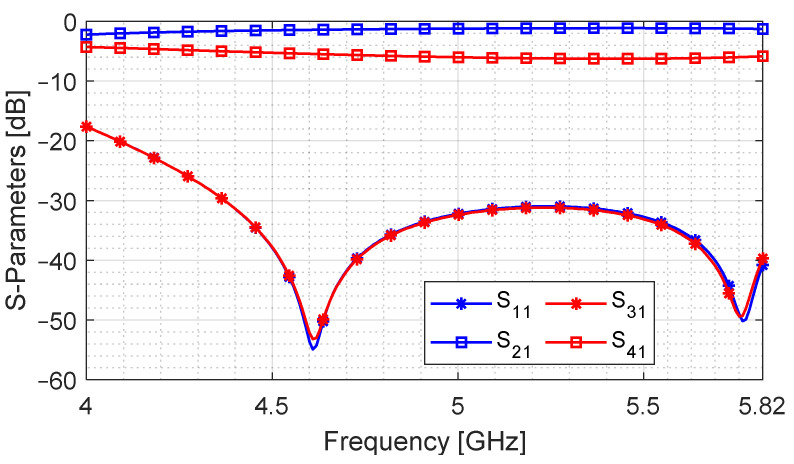
S-parameters of the slot coupler with reduced length. Design parameters are the same as in Table 1, and $L_{1}=53\;mm$.

Figure 12 shows all the S-parameters of the intermediate design. $S_{41}$ is decreasing from 4 GHz to 4.75 GHz, while it becomes relatively constant for the rest of the band. Furthermore, it should be noted that $\left|S_{11}\right|=\left|S_{31}\right|$, which is mathematically consistent.

### 3.2. Cross-Slot Apertures

**Figure 13:**
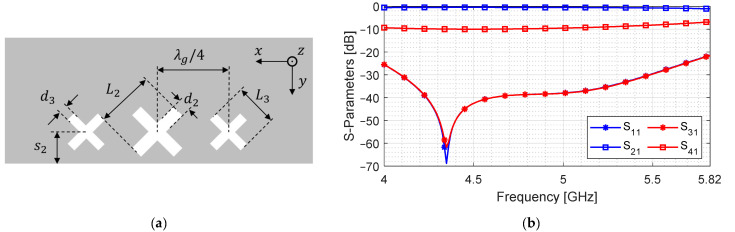
Design of cross-slot aperture. (**a**) Top view of broad-wall; (**b**) S-parameters.

As discussed in the previous section, a rotated slot can augment the slope of $S_{41}$ with a weakly coupled signal. Cross-slots typically provide weak coupling and are symmetric; therefore, we incorporate those in our design [28]. They can be added in the transverse direction to enhance the weak coupling of the slot aperture without increasing the length. We design three cross-slots that are $\lambda_{g}/4$ apart for improved isolation. The geometric parameters for this coupler are listed in Table 3, while the broad-wall and S-parameters are shown in Figure 13.

The design is tuned such that the coupled port signal is lower in the lower band and higher in the upper band ($S_{41}$ in Figure 13) for the same range of frequency; while the overall level is weaker than its rectangular slot counterpart. This is performed in order to perfectly complement the coupled signal of the slot aperture ($S_{41}$ in Figure 12) and achieve power equality with the least amount of fluctuation across the entire band

### 3.3. Combined Apertures

**Figure 14:**
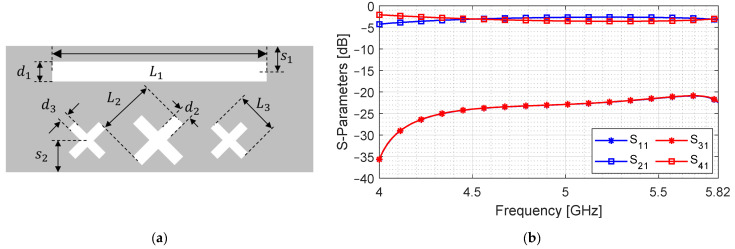
Design of the multi-aperture coupler. (**a**) Top view of broad-wall; (**b**) S-parameters.

The two types of apertures presented in the previous sections are placed on a common broad-wall without changing any geometric parameters. Figure 14 shows the broad-wall and S-parameters of the composite device.

**Figure 15:**
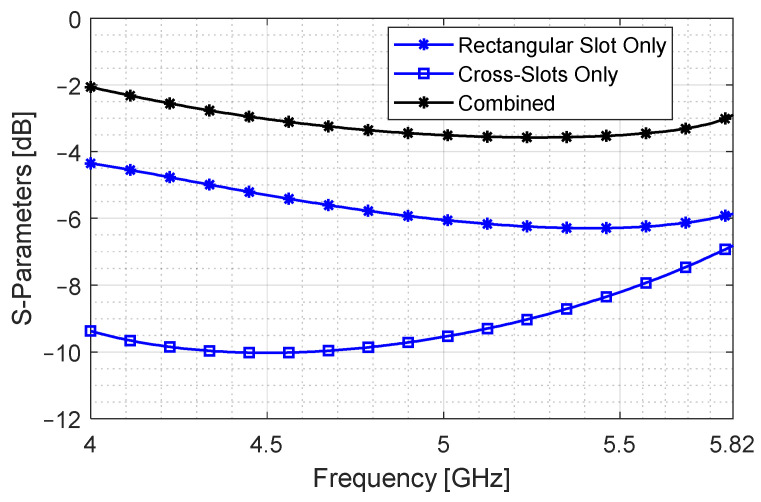
Coupled signal strength for rectangular slot aperture, cross-slot aperture and a combination of both.

It can be observed in Figure 14 that, through port ($S_{21}$) and coupled port ($S_{41}$), the signals come closer to −3 dB level for the same bandwidth. For better clarity, Figure 15 shows the $S_{41}$ for the three cases, where the coupling level of the rectangular slot is elevated to −3 dB.

Notice the rising slope of the cross-slot only aperture coupler in the higher band. This is performed to counter the dropping $S_{41}$ of the rectangular slot-only coupler. It is evident from the black curve that this approach increases coupling while stabilizing the coupled port signal. It should be noted that this is achieved with an aperture length of 53 mm, which is much smaller compared to the lengths shown in Figure 11b. This formulation shows that coupling level can be increased for a wide bandwidth without increasing the aperture length if the broad-wall area is efficiently utilized. This is a simple yet effective method that can greatly reduce the longer dimension of a coupling cavity.

## 4. Measured Results

**Figure 16:**
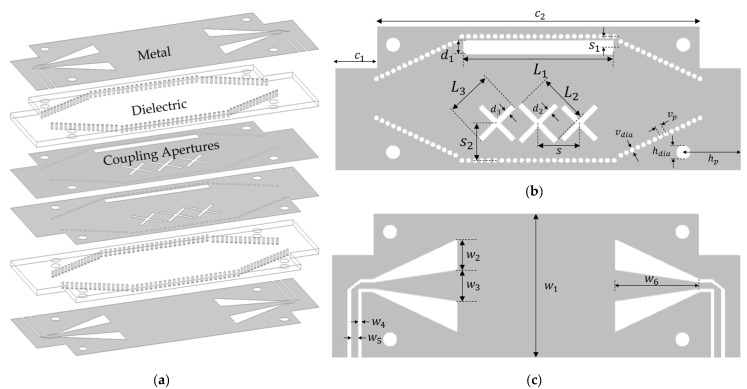
Implementation using SIW. (**a**) Exploded view of all PCB layers; (**b**) Top view of the broad-wall; (**c**) Top view of top and bottom layers showing GCPW-to-SIW transition.

**Figure 17:**
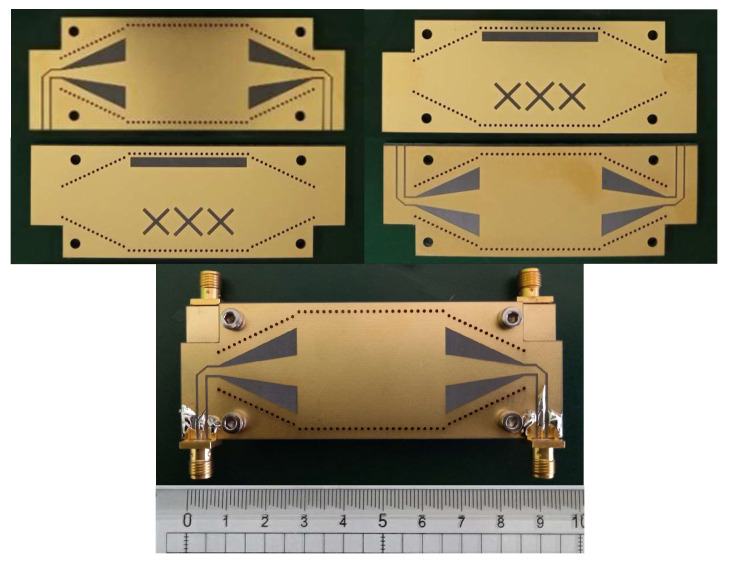
Fabricated PCB layers and assembled coupler.

For experimental validation of the stated method, a dual-layer SIW coupler is designed on Rogers RO5880 PCB laminate with a dielectric permittivity of 2.2 and loss tangent of 0.0012. The coupling cavity is fed using GCPW-to-SIW transition for seamless integration with the other components. The exploded view of the coupler is shown in Figure 16a. The top and bottom metal layers are identical and have the tapering that constitutes the GCPW-to-SIW feed. The metal layers in the middle are the broad-walls that have longitudinal and cross-slot apertures. There is a via cage to prevent signal leakage. All PCBs are cut at the corners to leave some room for connectors. Figure 16b,c show the top view of the broad-wall and the GCPW-to-SIW transition along with the geometric parameters, whose values are listed in Table 4. The fabricated PCB layers and the assembled device are shown in Figure 17. This design is optimized using HFSS and measured using an Agilent 5071B vector network analyzer.

**Figure 18:**
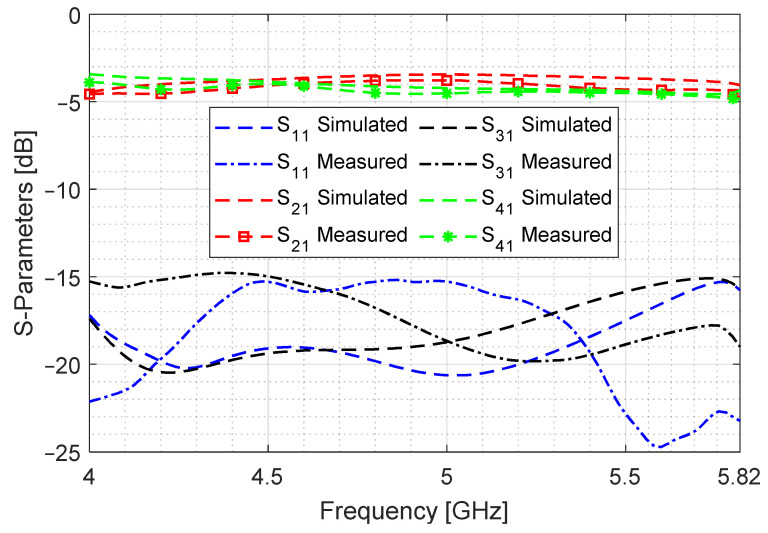
Comparison of simulated and measured S-parameters for the proposed design. Magnitude difference between $S_{21}$ and $S_{41}$ −4.3 ± 0.5 dB (37% FBW).

A comparison of simulated and measured results is shown in Figure 18. The return loss ($S_{11}$) and isolation ($S_{31}$) levels are below −15 dB. The difference in the through ($S_{21}$) and coupled ($S_{41}$) signals is under 0.5 dB for the entire band of 4 to 5.82 GHz, which amounts to 37% FBW. The measured signal fluctuates around –4.3 dB, which is close to the simulation. The difference of 1.3 dB (relative to the ideal value of −3 dB) is because of the adapter, SMA connector and cable losses, and it also includes the effect of the GCPW-to-SIW transition and the airgap between adjacent layers. This difference can be further reduced by using ultra-low loss connectors and feeding mechanism and by using multilayer PCB manufacturing technology. However, this method can be deemed acceptable, considering that the length of the coupling cavity is reduced and that the highest aperture dimension is the slot length, which is 0.63 $\lambda_{g}$.

**Figure 19:**
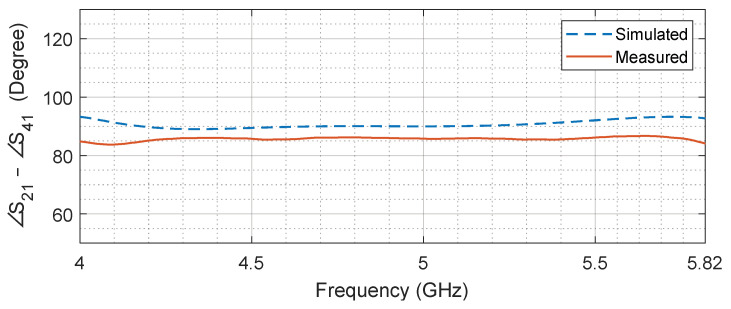
Simulated and measured phase difference between $S_{21}$ and $S_{41}$. The measured phase difference is 85.3 ± 1.4°.

In addition to equal power distribution, the coupled and through port signals are required to maintain a phase difference of 90°. Figure 19 shows the comparison of the simulated and measured phase difference. The simulated phase remains close to the ideal value for most of the bandwidth, while the measured phase fluctuates around 85.3° with a deviation of ±1.4°.

A comparison between this design and other −3 dB couplers is given in Table 5. For fair comparison, the length of the coupling aperture is considered to be the device length, as it will rule out the size of the feeding mechanism. Coupler_V is considered for comparison with [13] as it is smaller than Coupler_H. It can be generalized from this comparison that devices with a smaller length have reduced bandwidth, while the proposed method has the ability for longitudinal miniaturization without compromising the FBW. Although the proposed design does not have the inherent wideband capability of HMSIW, it is shown that this method maintains strong coupling for a wide bandwidth, even on a conventional SIW. An amalgamation of this method with a slow wave structure has the potential for further miniaturization, and this type of approach can be used in aperture-coupled systems.

## 5. Conclusions

The Bethe–Hole theory suggests multiple apertures in the longitudinal direction for wide bandwidth, while a rectangular slot aperture requires a higher length for −3 dB coupling. This complicates the longitudinal miniaturization of a wideband −3 dB coupler. This paper proposes to use two non-identical aperture rows and merge weakly coupled fields that can be obtained by a miniaturized rectangular slot and three cross-slots that are etched in the transverse direction. This method is experimentally evaluated by designing a dual-layer SIW coupler whose cavity is fed by a GCPW-to-SIW transition. The measured results show that the proposed method yields a device whose coupling strength is −4.3 ± 0.5 dB for an FBW of 37% while having the maximum aperture length of 0.63 $\lambda_{g}.$ This method can be used for the longitudinal miniaturization of a Butler matrix and other aperture-coupled systems.

## Figures and Tables

**Table 1:** Frequency and geometric parameters of the waveguide and aperture shown in Figure 2.

Frequency	a	b	$L_{1}$	$d_{1}$	$s_{1}$
4–5 GHz	42	11	9	9	6

Unit: mm.

**Table 2:** Frequency and geometric parameters of the waveguide and aperture shown in Figure 6.

Frequency	a	b	$L_{2}$	$d_{2}$	$s_{2}$
4–5 GHz	42	11	18	9	11

Unit: mm.

**Table 3:** Geometric parameters of the cross-slot aperture coupler shown in Figure 13.

a	b	$L_{2}$	$d_{2}$	$s_{2}$	$L_{3}$	$d_{3}$
42	11	21	5	11.5	18	5

Unit: mm.

**Table 4:** Geometric parameters of the proposed design.

Parameter	$L_{1}$	$L_{2}$	$L_{3}$	$d_{1}$	$d_{2}$	$d_{3}$	$h_{dia}$	$w_{1}$	$w_{2}$	$w_{3}$	$w_{4}$
Value	36	12	11	3	1	1	3	35	7	8	0.5
Parameter	$v_{dia}$	s	$s_{1}$	$s_{2}$	$c_{1}$	$c_{2}$	$h_{p}$	$w_{5}$	$w_{6}$	$v_{p}$	
Value	0.8	10	2.6	9	10	78	14	2	20	1.6	

Unit: mm.

**Table 5:** Comparison with other −3 dB couplers.

Ref.	FBW	Magnitude (dB)	$\mathrm{Length}\;\times \;\mathrm{Width}\;({\lambda_{g}}^{2}$)
[12]	25%	−4.1 ± 0.5	1.3 × 0.8
[13]	21%	Not Given	0.94 × 0.32
[14]	34.5%	−4.4 ± 0.5	1.39 × 0.47
[17]	28.32%	−3.67 ± 0.5	0.517 × 1.01
[20]	21.5%	−3.2 ± 0.3	0.6 × 0.26
[22]	8%	Not Given	0.78 × 0.71
[23]	35.3%	−4.4 ± 0.5	1.3 × 0.9
This Work	37%	−4.3 ± 0.5	0.63 × 0.53

## Data Availability

Dataset available on request from the authors.
